# Supplementary material for: Genome-wide identification and expression analysis of GA20ox and GA3ox genes during pod development in peanut
Source: PeerJ. 2023 Oct 26;11:e16279. doi: 10.7717/peerj.16279 (PMC10615029; doi:10.7717/peerj.16279)
Supplement: Table S1 [file peerj-11-16279-s001.docx]

**Table S1. Gene specific primers used in this study**

| Gene ID | Primer name | Primer sequence（5'-3'） |
| --- | --- | --- |
| AhGA20ox1 | AhGA20ox1/4F | ATCAGAGCAAGACCGACAG |
|  | AhGA20ox1/4R | TCCAATAAGGAAAGCCAAT |
| AhGA20ox2/3 | AhGA20ox2/3F | TTCATCAGCCCAAATCTCA |
|  | AhGA20ox2/3R | CTTGGCTTGGCTTAAACTC |
| AhGA20ox5/6 | AhGA20ox5/6F | TTGGCAGTCCTCAAGAGCG |
|  | AhGA20ox5/6R | AGCCCAAATATGGTGTCAACC |
| AhGA20ox7/8 | AhGA20ox7/8F | CCAACCCAACATTCCATCT |
|  | AhGA20ox7/8R | GCTTCCATGTCGCCAGAG |
| AhGA20ox9/11 | AhGA20ox9/11F | CACCAAATCCCAAGCCAATT |
|  | AhGA20ox9/11R | GGGTCGCCAGACAAGAAGC |
| AhGA20ox10 | AhGA20ox10F | CCAAACGGAAAGAAAGTAG |
|  | AhGA20ox10R | TCAAGGGTTGTGGAGTCAG |
| AhGA20ox12/15 | AhGA20ox12/15F | GGGTTGAAAGATAGATTGCC |
|  | AhGA20ox12/15R | GGTCCTGTCCCAAGTGTAA |
| AhGA20ox13/14 | AhGA20ox13/14F | AAGCCTCTGTCCATGTCCC |
|  | AhGA20ox13/14R | CATCCATCAAAGCGTGAGC |
| AhGA3ox1 | AhGA3ox1F | ACCCTGATTTCAAGTCCCT |
|  | AhGA3ox1R | AGAAGTAGTATTGATGGCGAGA |
| AhGA3ox2/3 | AhGA3ox2/3F | CGTGGGTTCTCCTTCTTCA |
|  | AhGA3ox2/3R | GGCTCCGTTATTCGAGTTG |
| AhGA3ox4/5 | AhGA3ox4/5F | GTCATGGAGATGAGAATGTCC |
|  | AhGA3ox4/5R | CGGTTTGCTGAATGTCACG |
| AhActin | AhActinqrtF | GTCATCGTCATCCTCTTCTC |
|  | AhActinqrtR | CATTCCTGTTCCATTGTCAC |
