# Supplementary material for: Genome-wide identification and expression analysis of GA20ox and GA3ox genes during pod development in peanut
Source: PeerJ. 2023 Oct 26;11:e16279. doi: 10.7717/peerj.16279 (PMC10615029; doi:10.7717/peerj.16279)
Supplement: Table S2 [file peerj-11-16279-s002.docx]

**Table S2. The information of AhGA20ox and AhGA3ox gene family members of peanut, *Arabidopsis*, rice and soybean**

| **Gene name** | **Gene accession number** | **Chrome** | **Length of CDS** | **Length of peptide** | **pI** | **MW（Da）** |
| --- | --- | --- | --- | --- | --- | --- |
| **AhGA20ox1** | LOC112755206 | 2 | 1119 | 372 | 6.14 | 42612.51 |
| **AhGA20ox2** | LOC112754423 | 2 | 1119 | 372 | 5.70 | 42603.44 |
| **AhGA20ox3** | LOC112728370 | 12 | 1119 | 372 | 5.70 | 42531.33 |
| **AhGA20ox4** | LOC112728437 | 12 | 1287 | 428 | 6.20 | 49169.01 |
| **AhGA20ox5** | LOC112791095 | 3 | 1140 | 379 | 6.16 | 42955.71 |
| **AhGA20ox6** | LOC112733062 | 13 | 1140 | 379 | 5.73 | 42927.69 |
| **AhGA20ox7** | LOC112794404 | 4 | 1140 | 379 | 6.76 | 43758.31 |
| **AhGA20ox8** | LOC112740586 | 14 | 1140 | 379 | 7.09 | 43671.10 |
| **AhGA20ox9** | LOC112802766 | 5 | 1149 | 382 | 5.80 | 43477.38 |
| **AhGA20ox10** | LOC112799975 | 5 | 1116 | 371 | 5.14 | 42256.14 |
| **AhGA20ox11** | LOC112751696 | 15 | 1149 | 382 | 6.27 | 43374.34 |
| **AhGA20ox12** | LOC112706011 | 8 | 1092 | 363 | 6.94 | 41469.38 |
| **AhGA20ox13** | LOC112711704 | 9 | 1137 | 378 | 6.16 | 42856.01 |
| **AhGA20ox14** | LOC112779497 | 19 | 1137 | 378 | 6.16 | 42909.95 |
| **AhGA20ox15** | LOC112767252 | 17 | 1092 | 363 | 6.94 | 41371.32 |
| **AtGA20ox1** | 828645 | 4 | 1428 | 377 | 5.77 | 43224.26 |
| **AtGA20ox2** | 835256 | 5 | 1358 | 378 | 4.90 | 33520.78 |
| **AtGA20ox3** | 830611 | 5 | 1323 | 380 | 6.90 | 43437.39 |
| **AtGA20ox4** | 842389 | 1 | 1568 | 376 | 7.14 | 43133.35 |
| **AtGA20ox5** | 841012 | 1 | 1810 | 385 | 8.04 | 43161.15 |
| **GmGA20ox1** | LOC100777224 | 3 | 1149 | 382 | 6.15 | 43484.61 |
| **GmGA20ox2** | LOC100775693 | 7 | 1191 | 396 | 6.50 | 44987.53 |
| **GmGA20ox3** | LOC100305378 | 9 | 1149 | 382 | 5.63 | 43363.39 |
| **GmGA20ox4** | LOC100803253） | 10 | 1098 | 365 | 5.64 | 41163.03 |
| **GmGA20ox5** | LOC100801187 | 13 | 1128 | 375 | 6.27 | 42797.56 |
| **GmGA20ox6** | LOC100787976 | 14 | 1047 | 348 | 6.37 | 39525.06 |
| **GmGA20ox7** | LOC100305379 | 16 | 1026 | 341 | 5.85 | 38535.98 |
| **GmGA20ox8** | LOC100816992 | 20 | 1152 | 383 | 5.85 | 43327.47 |
| **OsGA20ox1** | LOC4334841 | 3 | 1854 | 372 | 5.98 | 42255.71 |
| **OsGA20ox2** | LOC4325003 | 1 | 3123 | 389 | 5.73 | 42513.19 |
| **OsGA20ox3** | LOC4342505 | 7 | 2744 | 367 | 5.75 | 40494.75 |
| **OsGA20ox4** | LOC9269090 | 5 | 6929 | 445 | 6.70 | 47634.91 |
| **OsGA20ox5** | LOC4333459 | 3 | 2008 | 352 | 5.17 | 39293.06 |
| **OsGA20ox6** | LOC4336150 | 4 | 2123 | 300 | 5.51 | 32102.33 |
| **OsGA20ox7** | LOC4346321 | 8 | 2380 | 383 | 5.96 | 41831.59 |
| **OsGA20ox8** | LOC4337180 | 4 | 3640 | 326 | 5.34 | 35797.44 |
| **AhGA3ox1** | LOC112703711 | 7 | 1128 | 375 | 8.11 | 41693.86 |
| **AhGA3ox2** | LOC112707838 | 8 | 1059 | 352 | 7.30 | 40020.57 |
| **AhGA3ox3** | LOC112769088 | 18 | 1065 | 354 | 6.49 | 40322.80 |
| **AhGA3ox4** | LOC112709815 | 9 | 1086 | 361 | 8.07 | 40659.64 |
| **AhGA3ox5** | LOC112777276 | 19 | 1095 | 364 | 8.08 | 41025.03 |
| **AtGA3ox1** | 838125 | 1 | 1077 | 358 | 6.34 | 40161.81 |
| **AtGA3ox2** | 844374 | 1 | 1044 | 347 | 6.56 | 38782.41 |
| **AtGA3ox3** | 828256 | 4 | 1050 | 349 | 6.16 | 39210.76 |
| **AtGA3ox4** | 844373 | 1 | 1068 | 355 | 5.49 | 39152.51 |
| **GmGA3ox1** | LOC100818754 | 4 | 1026 | 341 | 6.62 | 38420.91 |
| **GmGA3ox2** | LOC100794325 | 6 | 1044 | 347 | 6.18 | 39059.39 |
| **GmGA3ox3** | LOC100776755 | 13 | 1059 | 352 | 8.52 | 39245.13 |
| **GmGA3ox4** | LOC100780857 | 14 | 1044 | 347 | 6.45 | 38768.46 |
| **GmGA3ox5** | LOC100798572 | 15 | 1062 | 353 | 7.72 | 39313.07 |
| **GmGA3ox6** | LOC100808546 | 17 | 1053 | 350 | 6.65 | 39126.85 |
| **OsGA3ox1** | LOC4337968 | 5 | 1155 | 384 | 5.95 | 41555.12 |
| **OsGA3ox2** | LOC4323864 | 1 | 1122 | 373 | 6.47 | 40572.22 |
