# Supplementary material for: Genome-wide identification and expression analysis of GA20ox and GA3ox genes during pod development in peanut
Source: PeerJ. 2023 Oct 26;11:e16279. doi: 10.7717/peerj.16279 (PMC10615029; doi:10.7717/peerj.16279)
Supplement: Table S3 [file peerj-11-16279-s003.docx]

**Table S3. The promoter region of *AhGA20ox* and *AhGA3ox* genes**

>AhGA20ox1-p

TTCGACATCTTCTTTGAATTTGGACTTTGCCACCTTTCTAGGATTCCATCCAAACCAACATCCCTCAACCATTTTTCAACATACCAATAATAATCACCAAATCAAAAAGTTTAACATAGTCATTAACTCAAAAAAATCAACATAACCAAGAATTTCAGTAATCAAATCAAATTAATCACGTACATAACCACAAATAAATCGACTTCATTCATTATTCTCACAAAACCGCCATAATCCATGTGTAATTTCTCATTTAATCTTATTGTTTGAGTAATTAATCAAATCATATCCATCCAAATAGTACAGCTTAATCCATAAGACTCGCATTATGCAAAGCTTAATCACAAAAATTCATTTTTTACATTAATATTTATTTATAACAATTTTTTTAAAAAAATAGATTTTAAAAAAACTCCTACTTCAATCGCGACTCAACTACGTAACAAACTCCTTTTTATTCTTATTTTGCAACAGCGGTATCAGTTTCGACTGTTTCTCGCAACAGCAACAACCACAAACACAGCATAAATCATAGTAACTCAATCTTGAGACATTAACGTCGCAAAATCCTCAACAAACTATAACAGAACACTAACATTGAGAATTTCAGAACAAAAAAACTTACTATACTTAAAAAAGAACCAAAGAATGGAGTAACAGTAGCTCCAATAGTCCTCACTAGCCAGAGCAGAACCTAAAAGAGACAACGCAACGCTAAAGAAAGGCAGAATAGCAACGGTAACTACTTCGGACAGCACTCACGACAACGCTTTCAGCAGCAACTACGACAGTTCTGTAAGATTAGGCGGCTGAACCCGACAACAATAATGTTAATTAGTGAGAACTAAATTCATAATTTGAAAAAAGACTCATTAATTTATAGGTACTATTATGAAGTACTTGATAAATATGTAAGTGATATCTTAATTTTATTTTTACACATAACAAAGATTTGTCTTTTGAAAAAGAAAATAGTTGTAACAAAAGATTTTTTTTGTGTGAAAAGGGTATCAATACTTATTATTTTATGTAAGGATAATTCAATATTTTAAACACAAATTCTTTCTATCATTCTACAAAAGTTAAGACAAGATATTGTACATTCATCTTATTTTTTGTAGTTTTGGTAGGTATTTAAACTAACAAAAAATATATAAAACTATTTTTAAGTATAAATACATCTATTTCAACTCAAAAATAAATAAAATATGTTAGTAAATTCCAATAGATAACAACGTTGGTTATATTATTTTAATTCTAAGAATGAATATAATACTAGAATATTAAATAGTAAGTAGATTTTAATAAAAATAGTTTTCTATGATATTATTTTTACTATGATAATTAAAAAAGTAATTCAAAGACAAATTTTATTTCATGTTATTAGATTGTACTTGAACAAATCAATTTTAATACAAGAGTAACAGAATTTAAAAAATTTTCGTGAATCACGATAAAATATCTATACATTCAATCATTAATATTATTTAAATATCTTTGAAAAAAAATAAGATTTTAGTATTTTAATTTAATTACAAACTATATATTACTATTAATAAATAATTATAGAATTTATCTATCATGTGTTATAAGGACATAAGTTAAAACTACAAATTAAAAATTTTACTAAAAATACAAAAAATTTAAATTTTTAATATATTTATTTTGTATTTATTAAATAAAAAAATTTAAAATCTTCTATTAATAGTAAGCTTATTATGTGTCCTTATGACACATGTTAGCTAAATTCTTATTATACTTAAAAAAAATAAAATTATTATTTATTAATTTAATTAATTTTATAAAATTTTATAACAAAAATTATTATATATTTTTTATTTAAAAAATAAAAAATACTTTACAGAATTTTAATTTGTATATGATCCCGTGTCCCCTCCACCTTTAATTTGCGTCATTTGCATGTACCCTAGTTCCCACTTCTCTGTATTCTATATATATGGGTACCGAGTATCCCAAAACATCATAGTAATCCTCAAATAACATTATTATTATTGTAGTGAGTTGCTTCACAATTGTCTTCGCATTTCAAAGACAAAA

>AhGA20ox2-p

GCAAAAGAGAATTGATAGGCACACAGTTTCTTCTTGTTATCATGCTATATAAAAAACATGTCTCAGTTGTGGTGGTTTGGTTTGGTTTTTGTTGTTTTCCATTGCAGGAATGCTATTGATTCCTCTTTATTCAAGCAATGGTTGCATAACTTGCAAAGTGAGATTGGGATTCTAGCTGATGGCACCTTGGCTCTGAGACAAGTTTTAATTCAGGTAATTGCATCATCAGTGTTTAAAAAGTGGATATGATTTCTGATAATTAGGCCTTGTTAAGTGGTAATTTAAGTAACTGACTAACCTGTTAGTCTTCTAGTCTTATTCATGTTCTCAAATCAAAATCAACAAAACTAGTTGGACTCTTTTTGGTGTAAACAGGGTGTAGACATGTTTGGAAAGCGCATTGGGTTTCTCAAATTTAAAGCGCATTGGGTTTCTCAAATTTAAAACTACTGGAATGTTGGATGATGACAAGGTTGATTTCGTTGGCACTGCAGTTCGTGAGGCTTCTCTCTCTCTCTCTCTCTCTCTCTCTCTCTCTCTCTCTCTCTCTCTCTCTCTCTCTCTCTCTCTCTCTCTCTCTCTCTCTCTCTCTCTCTCTCTCTCTCTCTCTCTCTCTCTCTCTCTTCATAGCTTAGTTGTGCCTCTGACAGGTTGAAGAGGAGACTGGCATAAAGTTCAAACTTGAAGACATGGTTGATCTCACTACTTTCTTGGATTCTTCAACAGGATGCAGATTTTTTCCCTCACCGGTATACCTTAAGCATTCTGCATCAACCTTATTGCTTTATTGGTTTTGCAAATAAATAATTTTGTCGCTTGAAAAAAGAGCTAATAATGCATCTGCTTTATAGTTGTTCAATAATTTAGTTTGGTATTTTGAGCATAGTTTAGTTTCGTGTTATTGTACTTTTAGATTTGCATTTACCAATCAGTGAGCATTCAATTCAATTTCTGGCTTTCTGTGGTTCAATCTAACAGTATTTTATCGTAGAATGTGAACCTGGTTTAATTTGGAATTGTTAATACATGCATAAATTAGATTATTTCTTATAATATTTTTATATATATCGTTTTTGTTGGTTCAATTTGTTCTTGATATTTTGCTGCAGGCTAGTATTGGAATGGATAACTTGAACGCTTAATGCACAAAGTGGAGCAGTTTTAGGTTGATTTTGATCTTTTACTAAGTATTTAGGTTGATGATCATCTCTATTAGTGTAATTTGATATATTATGAGCAGTTCTAACTTGTATTATTTCTGTATTTTTCTTCGGTTTTTAGTTTTGGAATTTGAACTCGAAATTTATTTTGTATTTGAGTAATAGTTTTTTAATGTATAAAACAATTATAGTAAATATCTCACTAATTATTGTTTGATTTGTCTTGTAATAAAAATTGCATACTATATTTGTAGGTTTGGTAATAAAAAAGGATTGAAAATTTATATTGTGTAGCAATGAAGATCAAGTAACAAAAAGAATTTTTTTTTTAATTTAACAAGGCTTTCGCCACGCTTTTAAAGCGTGGCTATATATTTTGCTATGGAGCGTGGCCGTATATCTACCTATCACCACGCTTTAAAAGCGTGGCCGTATATCCACCTATCGTCACGCTTTAAAAGTGTGGCCGTATATCCACCTATCGCCACGCTTTAAAAGCGTAGCCATATCTCCACCTACAGCCACGCTTTAAAAGCGTGGCTATATTTCCCACCTACAGCCACGCTTTTCCAAGTGGCAATTTGTAAAAAAGCGTGGCAAACAAAAAGCGTGGCAATAGGCTGCAAAAAGCGTGGGGATAGAGCAACCGCCACCCTTTGATAGGTCACCCTTTCAAAAGCGTGGCGGTAGCTCAAAAAGCGTGGCGAAAAGCTATTGCCACGCTTTTTTCAGCTTTTCGCCACGCTTTAAAAGCGTGGCAAAAGACGTGTTTTCTTGTAGTGATATGGGTACCAATATCCCAAAACATCATAGTAATCCTCAAATAACATTATTATTATTGTAGTGATCGAGTTCACACTTCACAATTGTCTTCGCATTTCAAAGACAAAA

>AhGA20ox3-p

TGCCATATCCATTGCATCTTTGTCGTTTTTAAGCACGGCCAATCCTTCCTCTAACCCAGCATTCACAGCTTTATACCACATCGCAGCGATATCCTTCTTCAAAAATCCCTGTCTACTCATTATTTCATAAACCTCAATCAAATTTCATTCATCTTCATCACAGTAGTCTACAATGGACGTCTCCCCACACAAATACATAACAGCACCTTCTTCAAGCCGAAACCTACCCTGGTGATAAATTTTTACACTAAAATGACTCATCTCTTCCGCTACTTTTTCTATTACAAGTTTCAAACATCATAAAACAAGGGTAATCAATGTGCAGGAAAGAAAGTGTAAATCAAAACATTCTACTAACATCAACAACCCTAAACCCTAACAACAACAATATTGCAATCATAGAAACCCTATGCATTAACATCTTTACTTGCATGAATTATTCCTTTGAAATGAAAATCAAATATAGATTGCACATACCTTTTCACTTTATGCCGTTTCAAAGTTTTTAGTACCAAGACGGGGCAATGCTCTCCTGTATGTAGGGAAAACTAAAATACGAACTCGCTTCAATCTGTCTTCTTCCAGTGTAATTGAGCTCGTCGAAAAACAGAGGAGAAAAGTTCGATAGGGGTTCAGGGAATGTGGAAGATGAAGTGTTACGTTTTTCTTTCTCTCATCTGAATGCAAGCATTTCGTATGCTAAAAGAAACATTATGTGCGAAGGAAAACTCGCGTCAATACACACATTTGGGGTTAGCAACGTTTATTGTTCACGTAGGCAACTCCATTAGTGACAATTATAGGACTTATCCCAAACGTTGGGGATAAAAATAATACTTTACTCTTTTTTTATAAAGTGTATAGTATTTTGACTTCAATTAAGGTGGCACATTCTAGCGCCAGTTTTTACATGTCCTTCTTCTCAATGTCATTTAAAAAAAAATTAAATGTAAATAATGAAATTTTACCATGATTAAGAGAGTTTTCATTATAGACAAAATATATTTGACGAATTTGTTAATTATTAATCATTCATGTCAGTTAATTATGGTGTATATTCAAATATATATAATATCATTAATATTGTTTTAAAATTTTATGTAAAACATTAATTATATATTATAATTCTTTTTGAATTAATATCATCATATATTAGCTAAATTTATATAATATGCTATAAGATATTGTAATAAAAAATGATCATGATTATTTCCAAATTTCGTTAGGTTGATAACATATATTGGTTGCATTTTCAATTTATAAAAATACTAGAACTATACATCTATATATTAAACTTCTAACTAGTTAATTAGTTTATAAAAAATATATTAATCTAAAACAATAAAAGTATAATTTATAATTATTCTGTCTTTTTCTTTTCAACAGGACTAAATATTTTTTTTATATATGTTCTTAAATAATGATGTAACTATATTCATTATTTCTTATTCAATTTTAAAATTCACTTATTATAATATTCACGGTATAAATAAAATTTTTTTATTATATTTTATATCAATTATCCAAATTTTAAAAATAACATGCTAATTAAATTAATTATTTTTTAATAAAATTATAAAAATATAAAGAAACAAAATCTTTTAATTTATTTATAAAAATATTATTTACACACTAAAATTAATCACTAAATTCAGTTATTATATATTTATAGTTTAATTTATTTTAATATATATTTTATATTAGTAATTAATTTTGATAATTAATTTCGGTGTATAGTTAGCACGATTGATTTATTTATTTATTTATTTTATCAAAGATAGGAGACTCCAACCCGCAACCTCTTAATTGAATACGAAGAGATTATACCATTTGAGTTATAAGCCAGGATTGATTTATTTATTGCCTCTAATTAGTTGCCATTAAATTAGTTGTCTCTATTCTCCATCCTGTTGCCACTTCACCCTTTAATTTGCATCATTTGCATGTACCCTAGTTCCCACTTCTCTGTATTCTATTTCTATATATATGGGTACCAATATCCCAAAACATCATAGTAATCCTCAAACAACATTATTATTATTGTAGTGATCGAGTTCACACTTCACAATTGTCTTCGCATTTCAAAGACAAAA

>AhGA20ox4-p

GCCAGGAGGGTGTGTTTCTTTTTCTTCCTACATATGTATTGCTGCCTATTCTTCCTCTTCCTCCTTCTTGATTATCTTCTCGCATTATTTTTTCAAACCTCCACATGGGTTGCTGCATCAGCAAATCCCAGACCCAAAACCAAAATCCATATCACCAAAATAATCAAACCCCCCCCAACAATACCTTACACCCTCTCTCCCCAACTCAAACCTTAACCAACAAATACCACAGCGGCAACCACCACAAGTGTCAGAGGAAGAGTCTGTGAAAGAGGTCCTATTAGAAACATCCATTGCCAAACGAAATCAAGTTCCAATTTTGAAGCCAGAATCGGACACCCTTCTGCCTCCTCTTCAAAACCCTGATGACAAAATCGAATCAAAGAACCCACATCCCATTCCCAACCCCATTATCATCAACAAAAAAGAAGGTGAAGTCTCAGAGGTAGTGTCTCAGCTCTCAGAAGAACCATGCAGCATCAGTGGAAGCTTCTCCACCGCTACCACAGTTACGGAGAAGAGAGAAAAAGAAGTAACCAGTAAAAGAAGCATCAGGGAGGGAACAACAACAATGATGAAGAATCATAAGTGGAACAGCAACAGATCCCCGTCGATGAAGCGCCCTCACGCCTGTGATGGCAATGTCGCCTCCGGGAGGGAGCGGAGTGTTGAGGAAGAAGGCACCAGAGTGGGTATTGGAGAGTGAAGAGAGAGAGAGAGAGAGAGAGAGAGAGAGAGAGAGAGAGAGAGAGAGAGAGAGAGAGAGAGAGAGAGAGAGAGAGAGAGAGGTTGAAGATAAGGGAGGGAGTGCCACCTAGGATCGGATTCCCACGAACCAAGCTCAGATCCAAGTCAGGGCACTTTTGTCACACAGAAGGCATCTATCATGGGTATAACTTTAGTTTCAGGCTATCATGGGTACAAATGTCATCGTTTTCATCTATCATGGGTACAAATGGTCATTTATTCTAAGATTTAACATGTTAGCGATAAGTGTATTGGCATAATCAGGCTACAGATTATCTGGAACAGGCCGAGCATGATACTGGTAACCTTATTGAGGATCTTAAGACACAATCCTTGATAAAATAGCTACTTTACAACGCTAAACTTCAAGCTGCATGTCCAGTTCCATTTGATGAAGCTAAGTTGTGGAAAGGCCGAGTGATAAATATATTGGTTTATAGGTCTCTCTGGTAATTGAAAAGAAAAAAAAGTCAATAATTAACTTGATTCTATATTTTCCTCTCTGTTATCAACATCCATCTGTTATCAACTTCATCTCGACCAAATTCTTTTCGCCTTAATTTTTTCCCTATTGCTCCAGTAATTTACAGATTCTGTGTTCTTTTAATGTTTATTTCAAATACTGACTCTCTACTAATTTTGAGAAGCTTATTTGCAGGACTTCATTTTCAATTATGGGGGTTTGTTTGTAGCACTTGTTTTTGTGACTAACTGGGATTGCAACAACGTGTCTCTGATCTTCAGCAGGTAACTGAGCTGTTGGTTTTCAAGTCTTTAGTTGGGACTTTGTTATCTATTTCATAAAGAGATTTTCGAGTCTTATGTTAGATTCAATTCTTCTCATACAGAGTTCAGAAATTGAGAACACAATTTGCACGTTTTTGTGTTATCATCGCACTCCCAGCTAAACAGCAAATCGATTCATTTATTATTATTATTATTATTATTATTATTATTATTATTATTATTATTATTATTATTATTATTATTATTATTATTATTAAAAAGTTAATAATATAGCAATCATCTAATATTTTACATTAACATATTTCAGAAGTCCTCGGAGATTTATTTTTTCAGGCTTTAAAACTTTTCAGGATAAGCTGGATGAGTCAGCACACAAGGTATTAACTGATTATCAAGCTGCTACAGTGACTCTCTTAGCGCTATTATCAGCTGCACCTGGTGATGTGAGTATTACTTGTCATGCTTTCTTTCTTGTTGCATTTTCTTCATCTATTGGCTAAGCTTGATTTTAGAAGAAATTATTTATTAGTTATGCACATATACTTGTAATGAACTAAGCTCAGCTTATTTGAGGGAAGGGGTGTTTTATAAATCAAGCTTTACATCAATTTTGGGTGTTCTATTAGACTAGTTCAGCTTCACATCAATTTTGGGTGGTTCACAACAACAGATGCCTGGTTTGGAGCTTGGATTGTCGCAAAATAGACATATTGGGGTAATAAATCCACAGGTTTTGACTCAGATTTACCAGCAAATGGGTCAGGCTAGGGTGCACCAAGACCATTAAGAACACCAACACCACCACCACCACCAGGCTTAGAGG

>AhGA20ox5-p

TCACTTCTTAGCCTATTAAAAAAAAGAGCAAAAACTTGCTTGTTTTCTTCTTTTATTTATTTTTTTTAAAGCGCAAACTTATCTACATTTTCCTTTTAAATTTAACTATTGAATTTAACTAACACATGTCCAATACAAGTTGAAGTTATTATAAAAATTTTTATTAAAAAATACAAAGTTGAGTCTTTATCTAAATACAAAATTATTTTTTTTATAAATATTACCTTTAATATATATTCTTTGGTTTAGATCGGAGCACATGGTAGCTAACAATTTACTACTCCTTTTTGAAATAATAGATGATATAATAAATAAAAACTAATTAACAATTTTTGTTGAAATTAAAAAATAATACTTAATTTGTATAACAAAAATAAAAAGGCACTTAATATAAAGCGAAAGAATAATGTCGGCTTGTTTTTACAAGTAAACAAAAGATCACCCAAAATATTATTTAAATACATTATTGATGTTTTAAGATTTCATTTTATTCAAATTAATTTTTATATAAACCGGATAGATCATAATGTTAGCAAAAGTATTTGATTTATCCTTCTCTTAAAACTTTATGATAAGCGTACTATTAACTTTCCCTTTAATTTTAAGAAATGCCAGGAAACAACCTTCATTTTATATTATAATTCTGGTATGATAGTAAATAACCAGAGTAATTATAATTTCGCACAGACTCACTCATTTCTTTGGAATTAGTGTAGATTTCTATAGTAAATAGTTAGGGTTGAAAAAAAAAATTACAAAGTTCGAAAAGAGTTTAATTAATAGTGTAAAGAGATATAATTAATTATTTATAATATTATTTTAAAAAAATCTAAAATCCATCCCGAAAAAATCTCTAAAAATAAGATGATTTTATTGAAAGTCACAAGACTAATTAATAATATGTGTTTAAAATTAAAGCCAAATTTTGATAAATACAGATGAAAGAATATAAGTGTAAAATTTTTTTTTAAAAAGATTTTATTAGAGTTGATTATGAAAAATTGGCTTATAGATTTTCTTTTAATTAATTTGTGTTTTGGACTTATTAAAATTATAAAATAATTTTTATAAAAAAAATTATTGTTTTCGACATATTTATTTCCATAAAAATTTAAAATAATTTATTAATAATAATGATGTACACTTAAAATATAAAATTTCAAATCTTTAAAAATATAAAACCTGAGAGATGAAAGAATATCAATAAAAAAGAAGAAGATATATACAGAAAAAGTGAAACTAAATTTTTTTTTTTACTAATATTAACTATTTTTTTAAATTTTTTTATTTATTTTAAATAATAAAACTTTAATTATACACATTAAATTATAAATTATAAGAGAACAATGCGAATGAATCAATTTTAAATATTATTTTTGACTTTTACATTGAAATAATTTAAAATTAAAATTTTTATTAAATTTTATTTTTGAAATACTAGAGACTGAATTATTTTTAAAAAAAAGTGATATATTGCTATTTGTATAATTTATTAAATTCATTAAATAAATATTGTAATGTTTATTATTGTAGCTTTGCAATAAAAATTCAATCAAAATATACTTAAATCAATTAGATGTACTCATTTGTACTCTTATATTTTATTACTTTATTTTTTTATTATCTTCGTATTCATTTGGTAAAAAAGTACATCTAGATCAATTTAACGTAAGTTCTTTTCTTCAATCCAAATGTACAAAATTTTTAATTTTCTCATTCACAATTTTTTTTAACTTTAGAAAGAATTAAAAAATAATTAAAATTTAAATTAAAATAAAATAAATATTAAAGTAAAAAATCTCATATTAAATTTTAAATAAAATTTTATGATATATAATCCCATAAATAAAATTTTATAATATATAGTCTCATAAATATTTACATAATACTTTGTAAAATCTAAAATAAATAAAATTATTCACTCGTTAAAAATTAATTCCTATATAGTAAAAAGAAAGAATTATCTTCCACTAGAATATCTCTATATATACGTGTGTTTATAGACTCTTTTCTGCAATTCCTACATCTATCGTTTTCTGTTTCTAGTCTTTTATTCCTCTTTCCGAGTTTCAATTCCTAATTCCCAACCCA

>AhGA20ox6-p

AGTTGAGTCTTTATCAAAATACAAAATTAAATTTTTTTTTATAAATATTACCTTTAATATGTATCCTTTAGTTTAGATCGGAGCTCATGATAACTAACATACAATTTACTCCTTCCATTTTATATTAAATGTCTCTTTAGTTATGCACGCATAACTATTAAAATAATCATTAAGGTTTACCAAACAAATCAGTAATAAAAAATATCTTTAGTATTATTTTCACTCTCCATTAATTATTTACTTTTTAAATTTTTTCTATTTAATAATAGATGATATAATAAATAAAAACTAATTAACAATTTTTCTTAAAATTAGAAAATAATACTTAATTTGTATAACAAAAATAAAAAGACACTTAATATGAGAAGAGTAATTTATACACCTTTTCAGCTTGTTTTACAAGTAAATAAAAGATCACCCAAAATAAATGTTGTTTTAAGATTTCATTTTCTTCAAATTAATTTTTATATAAACCGGATAGATCATAATGTTAGCAAAAGTATTTGATTTATCTTTCTCTTAGAATTTTATGCTAAGCGAACTATTAACTTTCTATTTAATTTTAAGAAATGCCAGTAAATAACATTTATTTTATATTATAATTCTGGTCTCATAAGTCTAAAATATAATATGTATTCTTATTTTGTCTGCACAGATTGACTCATTTTTTTGGAATTAGCATAAATTTTTATAATAAATAACTAGAGTTGAAAAAAAAAGTTGAAAGTTGGAAAAGAGTTTAATTAATAGTGTAAAGAGATAGAATTAATTATTTATAATATTAACTATTTTTTATTTTATTTTTAATTTATATTATCATTCCAAAAAATATCTAAAATCACATCCCGAACAAATCTCTGAAAATAAGATGATTTTATTGAAAGTCACAAGAGTAATTAATAATATATGTGTTTAAAATTAAAGCCAAATTTTGATAAATACAGATAAAAGAATAAGTATAAAAAAATTATTTAAAAAGATTTGATTAAAGTTGATTATGGAAAATTGGCTTATAGACTTCCTCTTAATTAATTTGTGTTTTAGACTTATTAAAATTATAAAATAATTTTTATCAAAAAAATATATAAAATTTATTGTTTTTGGCTTATTTTTTACAATAAAAATTTAAAATCAATTATTAATAATAATTATGTACACTTAAAATACAAAATTTCAAATCTTTAAAAATATAAAACATGAAAGATGAAAGAATATAAATAAAAAAGAAAAAGCTATATACAGAAAAAGTGAAACTAGAAAACTATTTTTTTTACTAATATTAACTATTTTTTTTAAATTATTTTATTTATTTTAAATAATAAAACTTTAATTATGCATATTAAATTATAAATTATAAGAGATCCACTCGAATGAATCAACTTTAAATATTATTTTTTACCTTTACATTGAAATAATTCGAAATTAGAATTTGTATTAAATTTATTTTTAAAATACTAGAGACTGAATCATTTTGAAAAAAAAAAGTGATATACTGCTATTTTTATAATCTATTAAATTTATTAAATAAATGTTATAATATTTATTATTGTAGCGTTGTAACAAAAATTCAATCAAAATATACTCAAATCAATTAGATGTACTCAATTGTACTTCTATATTTTATTATTTTATTTTTTTATTATCTTCATATTCATTTGGTAAAAAATATATCTAGATCAATTTAAGGTAAGTTCTTTTCTTCAATCTAAATGTACAAAATTTTTAAATTTCTCATTCACAATTTTGTTTAACTTTAGAAATAATTAAAAAATAATTAAAATTTAAATTAAAATAAAATAAATATTGAAGTAAAAAATCTCATATTAAATTTTAAATAAAATTTTATGATATATAGTCCCATAAATAAAATTTTATAATATACAGTCCCATAAATATTTACGTCTAAAATAAATAAAATTATTCACTGGTTAAAAATTAATTCCTACATAGCAAAAGGAAAATAATTATCTCCCGCTAGAATATCTCTATATATACGTGTGTTTATAGACTTCTTTCTGCAATACCTACATTTGTCATTTTCTCTTTCTAGTCTTTATACCTCTTTCCGAGTTTCAATTCCTAATTCCCAACCCA

>AhGA20ox7-p

AGTCTTTTTTCATGACCAACCTATGACAATATGTGACTCTACAAGAAGGTATTTTGGTGATCTCTGACAGACACAATGGTATCAAGGCTGCACTAGAAGCACTGGATAGTAGTTGACTACCTCCTCAGGCATATGAAGCATTTTGCATTCGTCATGTCGCCGCTAATTTCTCCCTCAATTTCAAAGGTCAGGATGCAAAACGGCTAGTTGTGAATGCTGCTTATACGAAGATTGAGGCAAAATTTGACTATTGGTTTGATGTTATGAGGACTGAAAAGCCACCCGACACATGCATCGATACAACCATGCATGCCAATGCGGTTGAATCCCAGCTATCATTGCCCATTTCATCAAGATTTGCCACAAATAGCAACCATCGAATATGAACCCGATTCGTACTCTTGTCACCTAACAGTTGAGTGGATAGCAGCATCATGATACAGGCACGTGCGCATATGCGAACAGTATGCTCATTCACATCGGCTAATAGCACCCTAAACCTCTCGTGGAATCATGTGAAGTGGACTGTCATCTACTTGACCTTATTCAGTGGTAGCAGCTTGCCAAATAACTCTTGAAACCACTCCCAAGCTACTCTGCCATCTTCTATAAATTTTTGAAGTCAGTGAGGCATCTACTAACAATCTTTCCATTGACAGAAAATCCCAACTGATATGCCACATCTTGCAGTGTGATGATGCACTCTTTGAATGGCATGTGAAAGGTATGCGTATTAGGACGCCACCTATCAATGAATACGTTGACTATAGGCTCATTCAACCAGAACCAACGATTGTTCAATCTAGCCAACTGGTACAAGCTAGCCCGCTTCCAAATAAGATATGATCCTTTTATGCATAAGCATATTCTATTGTCTTCGAATGCTGTAAATACACTTATTTAACTGCATCATGTAATAAAAATAACCACACTGTAATTAAATTTTAATACTTACAAGCCTAAACCATAAATTATTACCAAATATATCCTAATCTAGTGAAATAACTACGTAGCTATTATCTATCCAATCTTTTAGAATTGAGAATAACAAACATAAAAATTCTAACATATTAAAACCTAATTAATAAATTACTGATAATTCTAATATATACATTAACTAAATATTTAGTTTAGTTCTCTTAAATTTGTATGTTAAATCTTTTTTGAGTTTAATTTTTAATAAAGTTGAAAAATAAGTACTACTATCTACATACAAAAATAGAACTAATGTACTTTAGAACAAGTAACAAAATGTGCCATTTTTTAAATTTTAAGTCTAAATTTTGAATTTTTTTTAACTTTTAAATTTAAATCTTAAGTTTTAAATACTAAACTATAAATCTTAAATTCTATATTCTAATTCCTATACTCTAAATATTAGATTTTATATTCTAAACTATAATACTAAAAAGAAAACAAACTAACCTCTTCATTGATGCCTCCGACAACGTGGACAACGCCGTTGAGTTGGTACAAGTGGTCTTCGTCCGCTTTGGTTTTACTTGAATATGCACACTCCAAAGAACCCAAATTCAATCCCAGTCTCCTCCTCCCTCTCTCAAGAATTTCCTCTCTCTAACCAAATTTTCTTTTCACATCAAATGAAGGATCCGCTGCTAGCTATCGCGGTTTTATAGCCATCCTTCACATAAACCACAGCACCTTTACAACAATTGCATATTTGTTCATAAACTGCTATAGACAGTAGCGATTTATGCATGACACAGATCATTCAAAAATCGCTACATCTTCTAACAGTTTTGGCCAGCACGCTTTTCTACATAATCCGCTTCACACTCCAACAGATTACATTCAACTCAAAACCGCTACTCCCGATAACGACTTACATTAGATAAAGAAAAATTGTAAAAAAGTATGCAATTTCAAAAAATTGTAATCTAATAATTTTGGTTTCTAATCATTTTATTTAAGTACCTTGCTCAATTTTTTTTCTTTCAATAACAGAAACCAAACTACTATAAGTAAGAAACACAAATTCACTCCCTCAACTACCAAACCAAAGCTACTCTTCTAAAGTTTCAAAATTTGCCACTTTTAATCGCTTGTAACAAAAAAAGGTTCTAGAGAACGAAAACACTTTGTTTAATTCTTCATTTCTAAGA

>AhGA20ox8-p

GCAGTTTACAACAGTCCACATATTTTTTTATAAACCCCTATAAGCAGTAACGATTTATGCATGACACAATCGCTATACCTTCTAACAATTTTTGGTCAGTACGCTTTCCTACGTAATCCACTTTACAGTTCAACGGATTGTTGGGAATAATACACCATTCCCCCTTGAGAAAATACCTTTCACAAAGAAATAAAATAGACACAATCACAACACAATAATTTAACGTGGAAAACCCCAATTACTGGAGAAAAAAAACCACGGCCGTTGTCAAATGACAACCAGAGAATATCACTATGTGAAAATTGTTACAACACATAGACTTCTTTCTCTCTAACACCGGCACCCCAGTACACCCACACTCTCAAAGCAAATATTTAACTACACCTCACAATACTCTCTAATCAAAGAGTATAGAGGAAAAGAAAAGTCAGATACAAGCTTTAAGTGTTTTCGACTGGTGCAAAAAACATGGAGAACTTAGCCTCATATTTATAGCCTAGGCCACCCACTCCATTTGCTATCCTAAGTAATCTGGGACTAATTCAACTAAATCCTAACAATCTCCACCTTGATTGAAATAGTCACACATCTCCAGCTTCCATAGTTAACACTGACAATTCTTTGCTGCCATTGTCTATACCGACAATCATAGTTCAGAGAACTATCATACTCCACCATGAAAGTATACTCACTTGGAATTAGACCACTCCAAGCATTTCGCCTTGGTACAGATCGAAATCTTGCTGAAAATTCATGGTGCAACTTCCAAATTGGCTTTTCTTGGAAGTTCTTCAGCCATCGACATAACTCCGCCACACACCTTGCATCTCAACGCCAACCAATGCCCGTGTGCAATTGTGGACCCGATTGCCACAACTTATCCTTATCCATGGCAGTGCAGCAATACCATGAGGATACTTCTTATCTTCAACACCTTGTGCAAACCTAATTGTATCAACACATCCTTGACTTGTATTTGCCACAAGCCAAAATTGATTCTTCCATCAAATTTCTCTATTTCAAGCTTCACAGCACTTGAATATCCTGACATTGTTGCAACTGTATACTGGAATAGTATAACTCAAATGTAGATCGTGCACTAGGAAGGGTCCCCAGGAAAGAGAGGTGGGTCACAATGGACACACTTAAATACCAAGTCTTTCCTTAGCCAGAACCTTTCCAAACTGCACTCTCACAGTGTCACACTGCCTTCTAGCAACAACAACAGCAACCAAAGATCAACCTCAAGCCACAGGACAAAATTCTTTTCTGATGTGGAAGGTCAGACTAGGCTGCAACCACAAAGCATACTAAGAATAAATCCCACCGAACCGAAGCTCTAATACCATATGTTGGGAATAATACACCATTCTCCCTTGAGAAAATACCTTTCACAGAGAAATAAAATAGACACAATCACAACACAAGAATTTAACGTGGAAAACCCCAATTACCGGAGAAAAAAAACCACAGCCGTTGTCAAATGACAACCAGAGAATATCACTATGTGAAAATTGTTACAACACATAGACTTCTTTTTCTCTAACAACGGCACCTCAGTACACCCACACTCTTAAAGCAAATATTTAACTACACTTCACAACACTCTCTAATCAAAGAGTATAGAGGAAAAGAAAAGTCAGATACAAGCTTTAAGTGTTTTCGACTGGTACAAAAAACATGAAAAACTTAGCCTCATATTTATAGCCTAGGCCACCCACTCCATTTGCTATCCTAAGCAATGTGGGACTAATTCAACCAAATCCTAACACGGATTACATGCAACTCAAAATCGCTATACCTTATAGTGGTTTATACAAATAAAAAAAAAGTTATAAACAAGTATGCAATTTTAGAAAATTATATTTTGGTAATTTTAGTTTTCAATTATTTTATTTAAGTAATATGGTCTATTTTTTTTTCTTTCAATAACAGAAACCAAACTACTATAAGTAGAAACACAAATTCACTCCCTCAACTACCAAACCAAAACTACTCTTCTAAAGTTTCAAAATTTGCAACTTTTAATCGCTTGTAACAAAAAAAAGGTTCTAGAAATCGAAAACACTTTGTTTAATTCTTCATTTCTAAGA

>AhGA20ox9-p

AAGAATTTTATAAAATTAATTAAATTTAAAATTTAAATATATTTAATATTAAAAATAATAAAATTAATAAATAATAATTTTACTTATTTTCAAATTATAACAGTTCTTTATTAATAGTAATATATAATTTTTTATTAAATTGAATATTTACATTGAAATTTTATCTTTTTAAAGTTTTTTTTAAATAACATTAGTAGTTAAATTTATAAACATTTTCTCATGTTTTATGAATAAAATTTTTAATTTTTTATGTTAAAATTAATTTGAGCAAGTACAATTTAACATAAAATAAAATTTGTCGAAAAAATTTTTTTTATTATCATAATAAAAAAATTATGAAAAATTATTTTTATTAAAATTTATCTAGAATTATTTTATATTCTAATATCATATTCATTCTTAAAATTAAAGTAATACGAGAAATATTGGTATTTGTTAAAACTTACCAACATATTTTTTTTTGTTAGTTTAAATATTTACCAAAATTGTTAAAGATAAGATAAATGCATAATATCTTATCATAACAATGACTGATTTATTGATCCAAAATAATTTATAATATTTTTTTTATTTTAAGATAGATAATAAAAATTTTATTAAGAGAATATAGGATACAATTTATACTTATCTTTTATGCATAAATACAATTTTTATGAATGTTATTTTATCACTCAATTTCTCAAATTAAAATAAGCACATAAAGAATATAACTTAAACATTGAGAAGTTTCAACGTGTGAAGAGTTATGATAAATTAATTTATATGGAATAAAAATGATAAAATTTATAATAAAAATTGAATAAAGTGACAAATAAATCTCTGAGTATTTTATAATTTAGACAAATTAGCATATAAAGAAAAAAAGTACCAGTAAAGTTTTCTACGATAACAGATATGGATACACTACCTCCAAATTAATTCCTATAATGATGGTTGAAGGTCTTAATTTGAATATAACTTGTCCATGTCTGCTATTCTTGAGAATTTTATTGGTATTTTTTTAAAGATTAATATGTTCGAAGCGTAAATCTTTAAAAACTTATTTGTCACTTTATTCTAAAAAATTATAAAAACTTAAATTAACAGAAATAAAATAAAAAATAAAAAAGATAGAATGCAATAATAAATAATAATATTTTATAGTTTATAAAAAATCAAAGAATATAAATAACACATCATAGGAAAAAAAAAGTATTACTGAATAAAAGAGTAATTAGTAAAAATAATAAGATTCTCTTTTAATATATATGATTTTTTACAATAAACAAATTATAAAATACAAAAAATAGATAAAATAAAAGGAAACGAAAAAATTGTGTTTTTTTTTTTACAAATTGAAGAAATATCTACGATTATATAAGTGTTGGTGGACATATAACTATTAAAAAATAAATTACAAAAAAATATAAATTGACATATTAGATTTGATAGTAAAATAAAAATATAAGCAACGAATAAAAATCTAAAATTTAAAGTAAAAATATTTAAGATTAAGAAAGAAAACAAAGAAGAAGTTTTGATTAACAACGGCTCAAATATTAACATAACAATAAATTAAATTAAATAACATATATTTAAATTAATTAACATATATTTGAATTTATTAGTCAAAATACATTAAAAAAAACATACATGTACTTATCTTTTCATATTCAGTATTCAAAGAATCAAATGCATATTGCTTTTAATTAATAATGTCTTAGTAAAAGAATCAAATTAATATCGATTATAATAAAAGAAATCCTTACTCAAATAACTGTCTAAAATCAGAAATATTATTATGAATTAATAATGAAATTTTTTTAAAAATATTTGGAAAAATTTAATATTTTAATTAATTATTTACATAAAAATATATCCATGTGAATATAATATATAAATAATTAATATTTTTTAATTATAAATTTTTTTACTTTTCTAATCACAAGGAGTATCCTAACAAAATTGATGTTATTTTTTGAAGAGAATATTACTGTTTGTATGGAAGTGGTGCTTACAGTATTGAGTGAGTTTCACCAATTGTCCAATTGTGTCTACACAACTGAGAGAGAGAGAGAGAGAATGCCCTCTGCCTATATAAAGATTCGAACTTGTGTTGTGTGATTTCCCCAATAAGCGGCCTTGGCTTTTCCTTCATCCCCTCTCACAAACCATTTCTTTCTTTCAACATTTTTAAAAATTTCGAATTTGGAATATAAATATAGCTATACTTTTTCCTTGGTTAATGAA

>AhGA20ox10-p

ATAAACATTTCGAATTCTTTATAACTAACATTTTCTATTGTTACTGAAAATATAAAACAAGAAACATATATTATTCTTTAATTACTAAATCCACTCTTTAATGCTAGTCCATATTATTGGATTCATCATTCATGAACCATACCTATTAAAGAAAAACAAAATCCATTCGTGAATTGAACCTGGATATATATTTTGAAAACTATAACAATAAATTCTTTCTATCTTTTGTTGTAGATTATTCTTTATTTCTTTAATTACTACATTTGTGTTTGAAAAATTGATTTAAATTAGTCTAAAGTTATTTTATTTTATATTTATTATTTTTTAAAATAAATACATAATATCAAATATAATAAATATATCATTTTAGATGTTATATATAATTAATGATATTAAGTTAAATAAAATAATTTTAAATTATTATCTCACTAATATTATTGATGAAAAAAAATAGTTTCTAATAACAAAGATTGTGATTTTCATATAGAAAAATATTAAGAACTAATACATTTAGCCTAAAAGTACGTCAAATTTTGACTAAAACTAATTCAAATGTAAAGCCAAAACAAAAAAGGTGCCAATCACTTAGCATATTTTTTATTTGCAATTATATATGAAAAAGTATAGAAAAATAACGTTTTTTTAACAATCTAAATAATAGGTTAAAAAATCAGTTATTAACTGATTTTATGCAGACCAATCTCTTAATTTTCTCCTCACAAAAATCAATAGTATTCGTTTATAAATTCTTTTGCTAATAGAAGGATATAATTATTTATGTATTTTATTCAATAATTTAAATTATTTAGATAAATATTTTTATAATACTTGAATGAAAATATAATTATATATTTTTATTTATTAATTTAAATTTTTAAAATAAATAATTTTATTATAACTATTATTTAATAAACTACTATTTGTTATCATAAAAAATTTGAGCTCGAACAATTCTTAATTACAAAAATATAAATTAATTCAATATTCATATAAATTAGATTAATTTGACAAAATTATTCATAATATTCGATTGCACAATCTAACAAACTATTTATTTTAGTTAATTTTGTTAAACTAAATCAATTGAGTATAAAATTTAACATAAAAAATATTATTTGTATATTGAATTTAGTCACTAAATCAGTTGTTATATATTTGTGTATAAATATATGTGAATTTTAACATATTTTTAATATATATTTTATATTTTAATATATATTCTATACTAATTTTTTATTTTGGTATTCACCTAGTATAGTTGATTTATTATATAGAAAGTCTTGAAGACCACCAAATTTTAACAATTTTGATCATTAATTAGTCAACACAAATACTAAATTATTATTTCTAAACAGATAAACTTTACTAATTTATGTATCCAAACTTGGTAAACGTGGGTATAATTTATATTGATTTGTATATACAAATTTTGATAAATATAAGTATATTATGTATTTTATGTATACAAATTTTTTGTATATATATATATAAATTATTATTGATCAAATATAAATTCAAATAAAATAATAATATTTACTGAACATATAGAATTGCCAAAAAACAAATTTACATCTTTTAACTAAATAACCCAACAAAATATCCATTTTAGATAATTTATCAAACTAATTCCATGTTTTATGAAGATTGAATTAAATCATATTTTAGGAAATATATCTAGCTATATGTTCATTTAAGTTTTTTGTTCATATATATGTATATGCTTAGTCTCTAGAACTATGCAAAATGCAATTAACTTTGTGTCCATTCATATAGGGCTATGTCATTGCTGACCTCATCGCTCCTTGTTTCCAGTTATTAAGAAAAAATATAGGTTGAGCACTTTATTAAATTTTGATAAATAAGTAACTAGTAAAAGAAAAAATAAATAATTTTACATAATTAAAAATATTATTGATGACTATTTGATAACTATAAATCACAAAAGTTGTTGCTCGGTTATTAATTAGAGCTCCTCCATCTATATATATGTAACCAATCTACAAGAAACCACATATCTTCATTCTTCACAATCACAATAACAATAACAATAACA

>AhGA20ox11-p

GATAAATTAATTTATATGGAATAAAAATAATAAAATTTATAATAAAAAATTGAATAAAGTAACAAATAAGTCTCTGAGTATTTTATAATTTAGACAAATTAGCATATGAAAGAAAAAAATACCAGTAAAGTCTTCTACGATAACAGATATGGATACACTACCTCCAAATTAATCCCTATAATGATGGTCGAAGGTTTTAATTTGAATGTAACTTGTCCATATATCTGCTATCGTTGAGAATTTTATTGGTATTATTTTAAAGACTAATATGTTCAAAGCATAAATCTTTAGAAACGTATTTGTCACTTTATTCTAAAAAATTATAAAAACTTAAATTAATAGAAATAAAATAAAAAATAAAAAATAGAGTACAATAACAAATAATAATATTTTATAGTTTATAAAAAATCAAAGAATATAAATAACACATCATAGGAGAGAAAAAAGTATTATTGAATAGAAGAGTAATTAATAAAAATAATGAGATTCTCTTCTGATATATATGATTTTTTACAATAAACAAGTTATAAAATACAAAAATAGATAAAATAAAAAGAAATGAAAAGATTGTGTTTTTTTTTTTTTACAAATTGAAAAAATATCTACGGCCATATAAGTGATGGTGGACATATAACTATTAAAAAATAAATTGCAAAAAAATATAAATAAACATATTAGATTTGATATTAAAATAAAAATATAAGTAATGAATAAAAATCTAAAATTTAAAGTGAAAATATTTAAGACTAAGAAAGGAAACAAAGAAGAACTTTTGATTAACAACGGCTCAAATATTAATATAACAATAAATTAAATCAAATAACATATATTTAAATTAATTAAATTAAATAATTTATATAATAAATTAATTATAATTATTTGGCTTAATAAAGTAAATTAAATAAATATAAAATATATTAAAAATATGTCATGTAAATATTATAACACTTTTAAAAATTATTAATTAAAAATACATAAGATAAAAAAAATTAATACAAATTAAACAAAATCAATTTATTTATTTCAGTAAAATCAAATAATATAATTGATTAGTTATAGTTAATGTGGTCAAATAAAATATTAAATAATTATATATTTATTTCAATCAAATCAAATAAATGATTAATTATAACACTCTTACAAATTATTAATCAAAATGCATAAGTGGAAAAAATTAATACAAATAAAAAAAATAATTTATTTATTTCAGTAAAATTAAATAATTAACGTAATTGATTAGTTATAGTTAATGTGGCCAAACAAAAAATTAAATAGTTTTATATTTATCTTAATAAATCAAATAAATGATTAATTATGACACTTTTAAAAATCATTAATCAAAATATATAAAATTAAAAAAACCAGTACAAATTAAAATAAAATTAATTCATTTATTAAATCAAATCAAATAATTAATATAATTAATTAGTTATAGTTAATATAGTCAAATAAAATATTAAATAATTTAATATTTATCTCAATCAAATTAAATAAATAATTAATTATAATCCTTAAAAATTATTAGTCAAAATACATAAAAAAACATACATGTACTTATCTTTTCATATTCAGTATTCAGAGAATCAAATACATATTGCTTTTAATTAATAATGTCTTAGTAAAAGAATCAAATTAATATCGATTATAACAAAAGAAATCCTTACTCAAATAACTGTCTAAAATCAGAAATATTATTATGAATTAATAATGAACATTTCTTAAAATTATTTGGAAAAATTTAATATTTTAATTAATTATTTACATTAAAATATATCCATGTGAATATAATATATAAATAATAAATATTTTTTAATTATAAATTTTTATTTTTCTAATCACAAGGAGTATCCTAACAAAATTGATGTTATTTTTTGAAGAGAATATTACTGTTTGTATGGAAGTGGTGCTTACAGTATTGAGTGAGTTACACCAATTGTCCAATTGTGTCTACATAAGTGAGAGAGAGAGAATGCCCTCTGCCTATATAAAGATTCGAACTTGTGTTGTGTGATTTCCCCAATAAGCGGCCTTGGCTTTTCCTTCATCCCCTATCACAAACCATTTCTTTCTTTCAACATTTTTAACAATTTCGAATTTGGAATATAAATATAGCTATTTCTTTTTCCTTGGTTAATGAA

>AhGA20ox12-p

TTATATTCTATTTATTTTAATTTCAAAGTCACTCATTTTTAAATTAATTACATTTTATTTAACTATAAATTTTATTAAATATATACAAATTAAAAAGATAAACAAAAAATTTAATTAATCACAATTTATTTTTTTAATATATTATTTTATTTTGTCTTCATATAAGGTTGATAGTTGAAAATTGTTAGATGATATTTAGTTAAACTAGTCAAATAATTAAATTTTATATAATTATTTAATTATACCGAGTTAACCGGTTTAACCAGTGACCCACCGGTTAAACCAATAATCCAGTAACCTTACCGGTTCGATCACCAGTTCGATTCTGACAACTATGTTTAAAATAGTACAATACCAATGGCACATTCTAGAAACAATAATATATTTTTATTCGAGATAAAAATCTTAGGTCAGTTTTTATCGTAGTTATTATAATATATATTTGATTTTATCTATAACAATAGCCAAATTTTTATCTCAAATAAAAAATTTAGACATGTGACAATTTTATATTTATACGTAAGTTTCAATATTAATTGTAGTAATTTAACTATAAAATAGTGAGGTATTATTAATTAAAAGAAAAATTAAACACAACGAATGAATAATAGTAATTTCTTGATATATTGAAAATGGATGCAAGAAGTAGAAAACAATTTTATAGATAACTTAGGTCGGTATATAATTAGTTGATAACCATAACTACTGTGAAGTTTCTTATATATCTGTTATCCATATTCTTGATTTTTGTAAATCACTTATCGACATTTGACAGAGAATCAATTGAAAATCTAAACTCATAATTGTCCGTCAAATCAAGCAATTATTGTACTTCAACATAAACATGTTTAAAATAAAGTCAAAAGAAAACATTGTTTAAGGATTAAAAAAATAAAAAAATCTTGATCATTAGGATTTTTTGGACCCAATTTGATTAATTACTTTTCGAAGATCTCATTTTATCACCATCTTTTTTTCGGGTGACTTGTCAACATCCTAGACTTCACGTGTATTATAGTTGTTAAAATTAAACCAATAATCAACTTGATTAAGACATTAAGTTATTGGATTTGAGTAGTTTAATCGATAAATTATGAATTAAATTATGTAATCCAATAATAATTAAATAAATATATAAATTATAATAAAATTTAAAATTTAAATAATAAAATATATAAATAATATATAACATAAAATTTAAATTTAAAATTTATCTACAATTATACCGATATTTATCAAAATTTAAATATATTAAATAGAATATAATTTAATATTTATAAATTACATTATAAAATCATAATGTGAAATAAAATATAAAAAATATTATAAATTATAAATTTAATATTATAGTACAAACTATGGTGTATATATATAAGAGTAATATTAATAAAATTATAATTTATGGTGTATCTCATATTTTATTTTATATTATAATTTTATATAAGAGTAATATTACATGAGTAATAAAATTTATTACTCTAGTTAATATTTGATTAATATTTTAAATATTAAATATTAAATTTTAAATTTTAAATTTTAAATTATAATAATATAAAATAAAGCATTAATTATTCAGAATCAAAATCTTTTAAAGTAAAAAAATTATTATTCTTTTAAAATAAAAAATTAGTAAAATAATAACGTGAAAAATTAATCACTCTTAAACTAAGATAATAAATATACATTTTACAAAAATTATAAAATTAATAGTAATTAATTTTTTATTTTATCATTTTATTAACTCTCTTATTTTATTAGAGGATTTAAATTCAGTCAAATATTATCTAATACTATAAAAATATTAATTTACTAATATTTCTCTATACAATATAATTTATAAAATTATATTTTATACAATAAAATTTGCCCAATTTCGTCGGCAGCCAAAATAGTGAATATTGAACACCACAAGGTAACAATTACTACATTGAAAGCAGTAAGAAAATATAAAAGTAAACTCGCTCCACCATCTATATATATAAACACATGCCACAACCAAAAAGTTCTATATATATCTCCAACAATATTTAGCTATCACCATATAATTTGCTTGTGTAGTTGTGTATGTTCCCCTTTTTCTTCCTTCAATATTGATCACATACCTCAAATCATCGTTGACATTGGAAAAACATTA

>AhGA20ox13-p

TCGCAATTATGGAGGAAAGAATACCAAGAGAGGGTTGCTTAATGGATGGCTTAATGAGTAATAAAGCTGCCCAACTACAAGAACCTCATGAGCTTTGGGCCTTACTATTACAAGTATAAAGTGGGTTCCATGTATTTTCATCACAAACAGATAGGACTCTTGTTTTTCTCCTTGTTTTTTTTTTCCTCCCATCAAACGTAGAATCATGATTTGGGTAGTGGGTCATTTGTTGTTTTAGAAATTTTAAATTTGTTTAATGGTTAGTTTATTAGTCTAAAGTAAGTGTTTAGGAATTCGAATTTTATCTGGTGTATATAGTAATTTATTGGTCAACAATAAACCTTTAAATAAAGCTTCAATTCGCAATACCGTAAAAAAAAACTAAAAAGAGAGTACAATAATAGTATTTCCAAATCTAATTTAGTGGCACTAAAAGATTTGGTCTAATAGCATCTACGTGTAACTTGTTTAAAAAGTATATGAGATTCAAATCATGGTTATGACTAGAAAGTGATTATTATAGAACTCGTGAGAAGAAGGAGTATGATGCAGGAGCAAAAGCTACTTTGATGAATGATGACATGAAATTCTCTATATATGTGTGTCATTTTGTTGTTCTTGTAACTTTAGCTGACACCAGCAGAAAAAAACCTTAGTCCCTTTAAGTGTCTTCACCCTACTCTCCCATATATATTAGGCATTAGACACTCCTAATATTAGTTCCACAAGATCTGCCATTTGATCCAAATCTAATAATCCAATTGTTCCGTAAATTTCCAATAAGATTTATCATTAGTATTAGTATACTAACAAGTGTTATATTCATGATTCAGTTTTGGTAGAGACACATGATAGTGGCCTAGTGGGACTAGTCAGTGTTAGTAGTTAGTACTTGTGTTTTTGCCTTTCGTAATATGTATCAACTTACTGCAGTTGCTTCCCCTTAAAAAGGTTCAAGACCTCTCTCAAGCATACAATGAGTTGAGGGTGGGAAAGTGGCAACAAGCACTATGAAAATTAATAATCACACATTCTACATATGAAGTCCATCATAAGGGTTTTAAGACAGTCTAAAAGTACTTAATAATCGCTGTATCATTTAATAAATACTTGTACTTATAAGACCAATAGATAACTAAATCAAATAATAAAAATTATATATCATGATATTAGATGTACAAATATTTTTTTAATCAAATCTAATTAAATTGAGATAAGCCCAAACAAAAAAAATGTATCTATGCATCGTTGTTCTGCTTCTCTTTGTTTTTTAACATAAAAAATTTTGTTAAAGAGCACAAAAATTTAATAGTATAACCCAAAATTTTCATATAATATAAAAAATTTTGCATATAACACGTAAATTTTTGTTGTAAATGTATTTTATTAGTTGAGTGAGTTAATAGTTTGGGTATGTGATTTTAAAAAAAAAATTAATATTGTGAGTATTTTATTAGTTGAATGTCTAAGTTCTAAATACGTGATTCTTCATTTTTCTTTTTTTTGTATCGTAAATATTTTGTTAGTTTTTTTTGTTTATTCAAACGGTGTACAAGACGGGTTCGAACTCTCGACACTTACTTAAGCGGACGAGTGAGCTGTCAGTATATATCTTGTTAGTTGGGTGCTAATATTTTGAGCATATAATCCTTAAAATTTTTTGTATTATGCATAAAAAATTTCTATAGTCTAATTTTGAAGATGAAAAATAAAAAAAATGACAAAAATTTTAAAAAAGCAAGCATACAATAAACGAACTTAATTGGACTTGATTTAATAAGTACTTGACTGTTTAATTTTATTGTAAAACATATTAATCTTAGTAAAATTAACATTAAAATCATATCATTAAAAAAATATATTTATATAAAAATATTAAGATAATAATTGAGATACAATAGATGATTTAATATATTTGATTAAACTAGCTGATATAATACCCAAGAATATTCAAAAAACAACAAAAATGGCTTTGTAAAAAAGGAGTACTAGGGCACGAGTACTCCATGAAATTTGTACGAAAATAGTGAAGGTGTGGGTGCTTATATAAGATTCCAATATTTGTTCTACAATTTCCTCAACAAACGGCCTTGGCTTTTCTTTCATCGCATCTCTCTAACTCTTTCTTCTCCTTCTTGAATGCA

>AhGA20ox14-p

ATGGCAGTAAACTTAATACCAAAGCAGAATGAAAGAGACTTTGATGAATGATGACATGAAATTCTCTAACTCTATGTGTGTGTCATTTTGTTGTTCTGATAACTTTAGCTGGCACCAGCAGAAAAAAACCTTGTTCCTTTAAGGGTCTTTACCCTACTCTCCCATATATATTAGGCATTGGACACTCCTAATATTAGTTCCACAAGATCTGCCATTTGATCCAAATCTAATAATCCAATTGTTCCGTAAATTTCCAATAAGATTTATCATTAGTATACTAACAAGTGTTGTATTCATGATTCAGTTTTGGTAGAGACATATGATAGTGGGAGTAGTCAGTGTTAGTAGTTAGTACTTGTGCTTTTGCCTTTCGCAATATGTATCAACTTACTACAGTTGCTTCCCCTTAAAAAGGTTCAGGACCTCTCTCAAGCATACAATGAGTTGAGGGTGGGAAAGTGGCAACAAGCACTATGAAAATTAATAATCACACATAGGGTGTTGAAATCCAATCAGATCCAAATTAAACCACTCATCCAATTCAATCCAAATCAAAACCGATTAAAACCACACTAATTCAGATTTGATTGGATTTTATTTTTTACAAATCGCTAGATTGGATCGGATTTTGGATCTACTTTTCATAACCAATCCAATCCAATTCAAACCGCACAATGTACTATAATATTATTATTTTATTATTATACTTACAATTATATTTATAACATGTTCAATTTATTATACATTTTTCTATTATTCGTGTATTATTATTATTTAATAAATATTTTATGTTTGAAATATTATTTATTTATTTATTTTAACTAACCTATAATTTTATTTCTATTATTATGTTATCGTTGGCTTTTTAAGATATGTTAAGACTTGTTATGTCATTGTTGATTATTTAAAATTTGATGTTAAGACTTGTTATATGTATTTAATTTTTTTTATTTAAAAAACCGCAAATACAAACCGATCCAAATCGCTTGTAATCGAATCGGATCGGATCAGATCGAATTTTCAAAAAATGTTCATCCAATCCAAACCGCACCACACATAAATTAAACGTTCGAATTGGATGACTTTTTCCCTTAAAACCGAACCAAACCACACCGCAAACACCCCTAGTAACACATTCTACATATGAAGTCCATCATAAGGGTTTCAATACAGTCTAAAAGTACTTAATAATAGCTGTATCATTTAATAAATACTTGTACTTATATGACCAATAGATAATTAAATCAAACAATAAAAATTATATACCATGATATTAGATATACAAATATTTTTGTAACCAAATCTAATTAAATTAGGATAAGCCCAAACAAAAAAATGTATCTATGCATCATTGTTTCTGCTTCTCTTTGTTTCTTTAACATAGAAAATTTTGTTAAAGAGCACAAAAATTTAATGGTATAACACAAAATTTTCATATAATATAAATTTATTAATATAACATAAAAAATTTTGCATATAACACATAAATTTTTGTTGTGAATGTATTTTATTAGTTGAGTGAGTTAATAATTTGGGTATGTGATTTTTCAAAAAAAAAAAAATCGATATTGTGAGTATTTTATTAATTGAGTGTCTAGGTTCTAAATACGTGATTTTTCATTTTTTTTGTATTGTATATATTTTGTTAGTTGGGTGCTAATATTTTGAGCATATAATTCTTAAAAAATTTTTGTATTATGCATAAAAAATTTCTATGATATAATTTTGAAGATGAAAAATAAAAAAATGACAAAAATTTAAAAAACGTGCTTACAATAAACGAACTTAATTGAACTTGATTTAATAAGTACTTGACTGTTTTTCTTTTGTGACTAAGTACTTGACTGTTTAATTTTATTGTAAAACATATTAATCTTAATAAAATTAACATTAAAATCATATTATTAAAAAAATATATTTATATAAAAATATTAAGATAATAATTGAGATACAATAAATGATTCAATATATTTGATTAAACTACCTGATATAATACCCAAGAACATCCTCAAAAAACAGCAAAAATGGCTTTGTAAAAAAAGAGTACTAGGGAGGGAGTACTCCATGAAATTTGTATGAAAATAGTGAAGGTGTGGGTGCTTATATAAGATTCCAATATTTGTTCTACAATTTCCCCAACAAACGGCCTTGGCTTTTCTTTCATCGCATCTCTCCAACTCTTTCTTCTTCGTCTTCTTCTTGAATGCA

>AhGA20ox15-p

CACCCCCTAGTGCTGCCTTCTTATCTGTGCAACTCACGTAGAGGTTGGGAGAAAAGAACATATTAAATATGTTTCTAGAATGCACCATTGTTATTGCTGTTATTCGTAGACTATCATTTCTTTACCGTCAGTCACCAGATGATAAATCTTAGGTCAGTTTTATCGTGGTTATTATAATATATATTTGATTTTATCTATAACAATAACCAAACTTTTATCTCAAATAAAAAATTTAGACATGTGACAATTTTATATTTATATGTAAGTTTCAATATTAATTTAATATTCACAATTTAACTATAAAATAGCGAGGTATTATTAATTAAAAGAAAAATTAAACACAATGAATGAATAATAGTAATTTCTTGAGATATTGAAAATGGATGCAAGAAGAAGAAAACAATTTTATAGATAACTTAGGTCGGTATATTATTAGTTGATAACCATAACTACTGTGAAGTTTCTTATATATCTGTTATCCATATTCTTGATTTTTGTAAATCACCTATCGACATTTGACAGAGAATCAATTGGAAATCTAAACTCATAATTGTCCGTCAAATCAAGCAATTATTGTACTTCAACATAAACATGTTTAAAATAAAGTCAAAAGAAAACATTGTTTAAGGATTAAAAAAATAAAAAAATCTTGATCATTAGGATTTTTTGGACCCAATTTGATTAATTACTTTTCGAAGATCTCATTTTATCATCATCTTTTTTTCGGGTGACTTGTCAACATCCTAGACTTCATCACGTGTATTATAGTTATTAAAATTAAACCAATAATCAACTTGATTAAGACATTTACACTCCGTTTGAATCAAGAGAATACGGGAGGAAAGAAAATTTAAAATTAAACCAATAATCAACTTGATTAATCGATAAATTATTAATTAAATTGTGTAATTCAATAATAATTAAATAAATATATAAAATTATAATAAAATTTAAAATTTAAATAATAAAATATACAAATAATAGCATAAAATTTAAAATTAAAATTTATCTATCTATAATTATATTGATAGTTATCAAAATTTAAATATATTAAAAAAATATAATTTAATATTAGTATAAAATTATAATGTGAAATAAAATATAAAAAAAATTATAAATTATAATTTTAATATTGTAGTACAAAATATAGTGTATATATAAGAGTAATATTAAAATTATAATTTATAGTGTATCTCATATATATTTTATTTTATATTATAATTTTATATAGAAGTAATATTACATAAACAATAAACTTTATTATTTTAATTAATATTTGGTTAACATTTTAAATATTAAATTTTAAATTTTAAATTTTAAATTATAATAGTATAAAAATAAAGTATTACTCCATCGTAATCAAAATTTTTTAAAGTAAAAAAATTATTATTCTTTTAAAGTAAAAAAAATTAGTAAAATAATAATGTGAAGAGTTAATACCTCTTAAACTAAGATAATGAATATATATTTTTATAAAAATTATAAAATTAATAGTAATTAACATTTTATTTTATCACTTTATTAACTCTCTTATTTTAGAAAATTTAAATTCAGTCAAATATTATCTAATATTATAAAAAATATTAATTTACTAATATTTCTCTTTATACAATATAATTTATAAAATTATATTTTATACAATAAAATTTGCCCAATTTCGTCGGCAGCCAAAATAGTGAATATTGAACACCACAAGGTAACAATTACTACATTGAAAGCAGTAAGAAAATATAAAAGTAAACTCGCTCCACCATCTATATATAAAAACAGAAAAAGTCTAAGCACAAGCAACTTTATCAAATTTTAGTCAGCATGTAACCAACAAAGAAAAATGAATCATTGGATGAAATTTCATACCAATCTCATACCATTTAAATTATCATTTATGGCTATTTAATGGCTACAAATCACAAAAGTTGCTGGCCCTAACACTCCTCGTATAAACACATGCCACAACCAAAAAGTTCTATATATATCTCCAACAATATTTAGCTATCACCATATAATTTGCTTGTGTAGTTGTGTATGTTCTCCCTTTTTCTTCCTTCAATATTGATCACATACCTCAAATCATCGTTGACATTGGAATAACATTA

>GA3ox1-p

GAATTGAATGAGATAAATCATAAATCATAACACAACTTGGAAGCCTTTCATTTAAATTTCTTTATACTTGAGGACAAGACAAAGGCACATGTTAACGTAGGGGCCCTTACTCTTTTCTTGGAATTTCAATTATTTACAACATTAGTATATATATTTAGTACAATCTAATAACAATATACATGTCTTAATTATAAGATTAGAGGATTGGATTTCACTTTCTTTTATTAGTGTGTGCAATATGTCTTGAGGACAAGATAGTCCTTTCCCATTGTATTGTTCATACCTACTAATCTACATGTATAATTTGGTTTGGGGTAAGGGGAAAGTTTGTCGCCTTGTGGCCTATGATCTCTATATATGCTATCTTGGCACAATCATGTTGTGCTCACCTCATCATCCTTTTTATTTTTTTCTTTTGGTACTATGTTTTCTAATTGCAATAAGTGTTAGGATTTGGTTGAATTAGTCCCACATTGCTTAGGATAGCAAATGGAGTGGGTGGCCTAGGCTATAAATATGAGGCTAAGTTCTCCATTTGTTTTTGCACCAGTCAGAAACACTTTAAGCTTGTATCTGATTTTTCTTTTCCTCTGTACTCTTTGATTAGAGAGTGTTGTGAGGTGTAGTTAGATATTTGCTTTGAAAGAGTGTGGGTGTACTGGGGTGCCGGTGAGAGAAAGAAGTCTATGTGTTGTAACAATTTTCACATAGTGATATTCTCTGGTTGTCATTTGACAACGGCCGTGGTTTTTTCTCCGGTAATTGGAGTTTCCACGTTAAATTCTTGTGTTGTGATTGTGTCTATTTTATTTCTCTGTCAAAGGTGTTTTCTCAAGGGGGAATTGTGTCTTATTCCCAACAAGTGGTATCAGAGCTTCGGTTCGGTGGGATTTATTCTTAGTATGCTCTGTGGTTGCAGCCTAGTCTAATCTCTTTGGTTGCTGTTGTTGTTGCTGGAAGGCAGTGTGACTCTGTGAGAGTGCAGTTTGGAAAAGGTTCTGGCTAAGGAAAGACTTGGTATTTAAGTGTGTCCATTGTGACCCACCTCTCTTTCCTGGGGACCCTTCCTAGTGCACGGTCTACAGTTGAGTTATAATATTCCAGTATACGGTTGCAACAATGTCTGGATATTCAAGTGCTGTGAAGCTTGAAATAGAGAAATTTGATGGAAGAATCAATTTTGGCTTGTGGCAAGTACAAGTCAAGGATGTGTTGATACAATCAGGTTTGCACAAGGCGTTGAAGGAGAAGATCTCTGGTTGCTCTCTCTATGAGAGAAGATGATGTTCTCTAGAAGACAAGAAGTATCCTCATGGTATTACCGCACTGCCATGGATAAGGATGAGTTGTGGCAATCAGGTCCACAATTGCACACGGGCATTGGTTGGCGTTGAGATGCAAGGTGTGTGGCGGAGTTAGGTCGATGGCTGAAGAACTTCCAGGAAAAGCCAATTTGGAAGTTGCACCATGAATTTTCAGCAAGGTTTCGATCTGTACCAAGGCGAAATTCTTGGAGTGGTCTAATTCCAAGTGAGTATACTTTCATGGTGGAGTATGATAGTTCTCTGAACTATGATTGTCGGTATAGACAATGGCAGCAAAGAATTGTCGGTGTTGACAATGGAAGCTGAAGATGTGTGACTATTTCAATCAAGGTGGAGATTGTTAGGATTTGGTTGAATTAGTCCCACATTGCTTAGGATAGCAAATGGAGTGGGTGGCCTAGGCTATAAATATGAGGCTAAGTTCTCCATTTGTTTTTGCACCAGTCAGAAACACTTTAAGCTTGTATCTGATTTTTCTTTTCCTCTGTACTCTTTGATTAGAGAGTGTTGTGAGGTGTAGTTAGATATTTGCTTTGAAAGAGTGTGGGTGTACTGGGGTGCCGGTGAGAGAAAGAAGTCTATGTGTTGTAACAATTTTCACATAGTGATATTCTCTGGTTGTCATTTGACAACGGCCGTGGTTTTTTCTCCGGTAATTGGAGTTTCCACGTTAAATTCTTGTGTTGTGATTGTGTCTATTTTATTTCTCTGTCAAAGGTGTTTTCTCAAGGGGGAATTGTGTCTTATTCCCAACAATAAGTACTAGCAAACCATCAATTGCCATAATATTTATAACACTCTAGTCTCTTGTTCCTACCCTAACAACATATACATATAATACTAAAGAATTAAAT

>GA3ox2-p

CAAAATTTCATCACTTTTTTTTTCTTTGGAAAATCAATAAAAGGGATTTTAATTAAGTCTCTCAGCATGGTTTAATTATTTTCCATTAATTATTACAAAACTCAAATTTAGTTTGAATCTCATAGTTAAAGGAAAAAAATATGGTTTAATTATTTTGTTGGTCCTTATAGTTTTTTAAAATTTTTAGTTAGGTCTCTATATTTTTTTTCTTTTTAATTGGATCTCTATACCAATTTTTTTTCAATTAGCTCTCTCTTAATAGTAATTGGCTTAATTTTATAGGGACTTAATTAAAAAAAATAGTGCAGGAACTTAAATAAAAAAAATATAGAGATTCAATTAAAAAAAATTAGTGTAATAAGGACTCAACTAAAAAAAATACAAAGATCTAATTAAAAATTTTACAAAATTATAACCAACAGAATAATTAAACTAAAAAAATATCTATTCCCCTAATCAAATCCTTGTTAGTTAGTATATTTTGATGTTCTAAAATACTCTTGCAGTTTTTTGTTTATACTCCGAACCATTTAATATCTAAACTCCAAAGTAAAAATTCTCCATAAATTATTGGTATGGAATATTGCGTATTAAATATATTAAATAAAATATAATAATAAGAACTTTATAATGCAAGATGTGCCCCTAGCTAGATCTCTTACTTTTCCAAGTATGCAAATAAGACAAATACATGATAAGCTTGAAAAAGAAAATCTTATAAATATAAAAAGGAATATATATATATATAGATCCAAATCAATTTATTGTATAATTTAATAATGATTTTTTATTATTTTTTTAAATCATGTCACTATTTCTTTTCTTTGTTTTTAAATTATGCACCTTGATGTAAATCAAATGTCTGTTAAATTCTTTGAATTTTGTCGTATATGGTAGCGCCAAGCTAATTATTTTATTGCAGCAACACGACAAAATGCCTGCCTCTTTTTTGTCAATAAAAGCCAAAAGCAAACAAAAAGAAATCCTCTCAAGATTTCAGTTTATTTTTTAAATAACACTATGTGTACATCAGTCATATATTGATTTTATATAATTTAATAAATTTTCCCTAAAAAATTGTTTATTCACAATTAAATTAATATAAGTAATGAAAAGATGAAATTTCTATATATGGTTGAGCTTTAATTTAAATATGTTTTATAGGTCCTATAATTATTATGTATAATTTTTATTTAGTCCACTCTTTTTTTTTTGGCTTTTGAAATCTCTCTTTTTTTCCGATTAATTATTTTGATTTTTTTTGTCTGCATCGTCTAAATTTATAGTTAAATTGTAAAGATATAAAAACTAAAAATTTTTGCTGTAAAGATGAAAGAAAAAGTTTTGATATTTACAGGACCTAAATCAATTTTCTTAGAAAACCAATTTTTCTCAACAGACAATTAGCTAACATTTATAAAAAAAAAAGAAAAAAAACTAAAACAAGGCAAAAATGTTATTCATATTATTTTGTATAAATTTATATATCCTATTATTGTATTGAAACCATAAGCGTAGAGATAAGTTTTTATGTTGATTCGTTTACATATTTTTTCTATTGGCTTATGGTGATGAGGTATAGTGAAAGCTAGCTTGTGGTGGAGAAAATAAAATTGTTCCCTTTTTGTTTTTATTGAGTAATACTTTTTTCAGAATAACTTGTTCTTTTCTTTGTCTTTTTCAACCATATTATTCTTCTCATTTCTCAAAATCCTAAACCGACACAACTTAGTCCAATTGCCACCCCCTTACGACAACTGTACACAATTTAATAATAATAATAATAATAATAATAATAATAATAATAATAATAATAATTAAAATTTTATATAAAATTTTCAATTTTTTCATATGCATATCTCTAGTCCAAGACCACTAATTAAGAAGGAAAAAAAAATCCGCCACAAAAAATGATACATTAGTATCTCACATTGTTTTAATATTAAGAATAATCTAATTAATCTTAGCATAATAGATTTGATATGCTATCTCCAAACATAAGTAATTATGATATAAATACATATGGTCCTAGGATGATTAAATCTCTATAGTAAACCAATCAAAGTAAGAAGCAATCAAATTAAAGCCACCCTAAAGTTTTGAAAAAAATAATAAT

>AhGA3ox3-p

GGTCTTTATACCAAATTTTTTTTCAATTAGATCCCTCTTGATAGTAATTGGCTTAATTTTATAGGGACCTAACTAAAAAAAAATAGTGCAAGGACCTAAATAAAAGGAAAAAAAGTGTAGGGACCCAATTAAAAAAAAAATTTGGTGTAAGAACTCAATTAAAAGAAAAAAAATATAAAGACCTAATTAAAAATTTTACAAAACTATAAGAACCAATAGAGTAATTAAACCAAAAAATATCTATTACCCTAATCAAATCCTTGTTAGTTAGTACATGTTGATGTTCTAAAGTACTCTTGCAGTTTTTTTTTTTATACTCCGAACCATTTAATAATGCTTTTTTTTAATAATAATAATTATTTTCAAAATATATATTACATTTAATATCTAAACTCTAAACTAAAAATTCTCCATAAATTATTGGTATGGAATATTGAGTATTAAATATATTAAATAAAATATAATAATAAAAACTTTATTATGCAAGATGTGCCCCTAGATCTCTTACTTTTCCAAGTATGCAAATAAGACAAATACATGATAAGCTTGAAAAAGAAAATCTTACAAATATAAAAAGGAATATAGATCCAAAATAATTTATTGTATAATTTAATAATACTTTTTTATTATTTTTTCAAATCATGTCACTATTTCTTTTCTTTGTTTTCAAATTATGCACCTTGATGTAAATCAAATGTCTGTTAAATTCTTTGAATATTTGTCGTATATGGTAGCGCCAAGCTAATTATTTTATTGCAGCAACACGACAAAATGCCTGCCTCTTTTTTGTCAATAAAAGCCAAAAGCAAACAAAAAGAAATCCTCTCAAGATTTCAGTTTATTTTTTAAAGATTTAATTACTCTACTTATCTCTATAGTTTTACAAAATTTTTAATTAGATCTTTATACTTTTTTTTTTAATTGAGTCTTTACACTAATTTTTTTTTAATTGGGTCCTTACACTTTTTTTCCTTTTATTTAGATCTCTGTACTAATTCTTTTTTTTAGTTGGGTCCCTATAAAATTAAACAAATTACTAGTAAGAGGGACTTAATTAAAAAAAAATTGGTATAGGGACCCAATTAAAAAAAAATATAGAGACCTAATTAAAAATTTTACGAAACTATTGGGACCAACAGAGTAATTAAACATTTTTTAAATAACACTATGTGTACATCAGTCATATATTGATTTTATGTAATTTAATAAATTTTCCCTAAAAAATTGTTTTTTACATATAAAAAATTTAACATCTTAGTATATATTTAAAATTTTAAATATATTCACAATGAAATTAATATAAGTAATAAAAATATGAAATTTCTATATATGGTTTTGATATTTACAGGACCTAAATCAATTTTCTTAGAAAACCAATTTTTCTCAACAGACAATTAGCTAACATTTCTAAAAAAAAAAAATAAAACAAGGCAAAAATTTTATTCATATTATTTTGTAGAAATTTATATATCCTAATAGTTTTATATTATTGTATTAAAACCATAAGCGTAGAGATACGTTTTTATTTTTATTGGTTTACATATTTTTTCTATTGGCTTATGCTGAGCTATAGTGAAAGCTAGCTTGTCGTGGAGAAAATAAAATTGTTCCCTTTTTGTTTTTATTGAGTAATACTTTTTTCAGAATAACTTGTTCTTTTCTTTGTCTTTTTCAACCATATTATTCTTCTCATTTCTCAAAATCCTAAACCGACACAACTTAGTCCAATTGCCACCCCCTTACGACAACTGTACACAATTTAATAATAATAATAATAATAATAATAATAATAATAATAATAATAATAACAATAATAGGATTATAAATTATGAAGAAGAAAAAAATTATACATTAGTATATCACATTGTTTTAATATTAAGAATAATCTAATTAATCTTAGCATAATAGATTTGATATGCTATCTCCAAACATAAGTAATTATAATATAAATACATATGGTCCTAGGATGATTAAATCTCTATAGTAAGAAGCAATCAAAATTAAAGCCACCCTAAAGTTTTGAAGAAAAAAAAAATAAT

>AhGA3ox4-p

AAATTATTTTATATATTTATTTATATATATATATACATATATTGATTCATATATTTTTAATATATATATATATATATATATATATATATATATTGTATTTTAATATATATTTTATATAAGTGATTAATTTTTTTATGTACATATAACTAATTTTTGCATTTTTTTTTTCAGAAATTACACTAATGTATGTAACAATATTTACACTCTTTATTTTTTAATAGAAGAAAAAAATTATTTATTTTTTAAAAATTTAATGTAAAAGAAAAATAAATACCCCTACCTTAATTAAAAAGCACCGTAGCATATGGGGATCATTCATAATGAAAGTTCAACAATAATAATTGACTATACTTTAAAGTAAGATTATTATTCTATTTTAAAAATAATATGTGATATTTAAATATAGAAATCATAGAGTTTATTTATTGAGGAGTGCTACACATACAAGTTATTTAGTTTACAAGTCATACAAGTTGTGAGAAACTTAACAAAAAGCACGCTGACGCTGACCCATATACGATTTTCATGGCGTGTTTCGCGTTCTTCTTTCTCCTCCTCCTCTTCCTTCTTCTTCTCCTTCTTCTTCTCCTACTCTTCTTCTTATTCCTCCATGAAAACGAGTTTCTTGCCAAATTTGATCTCCTTCGTGCGTTCTCGATCTCTTTGCCTTTTCATTCGTTATTTTATCTGCGTTTATAATTGGTATTCTTGCTTCATTTTTTTCGATTTTCATGGTTTCTGAAATCAAGTTTTGAAATCGTTTTGAAGATAATGGAACTTCAGAAATACACCCGAACGATTACAGAAATACACCCAAACAATTACAGAAATACACCAAAATGATTACAGAAATACACCCAAATGATTACAGAAATACACCCAAAGGATTATGAAAATACACCCAAAGGATTTAAAAAATACACCCAAAATTCGTTGAAGTACACCTTATGCATAATTCAGAACTCTTTCTCTTTCTCCTCCTTATTTTCTACTGCTTCTTCTTCTTCAAAAATGATTTTAGAGCTTGATGTCAAAAAATAATGAAAATTGAGAATAACGAAAAAAGAAAACAGAGAGAAAAATACGTAAATAAAGAAGGAGAAAGAAAAGACAAGAAACGAAAGAAAAAAAGAAGAAGAAGAAGAAGAGGAAGAAGAACCTGCAGCAAAAAGAAGAATGTGCAGAAGATGAGGAGGAAGAAGATGAAAAACCTGCAGTCAAGAAGAACGAGGAGAAATATGACGATGAAAATGAAACACTTCAAAAACGAAGAAGAAGAGGCACGGAAAAAGAAGAAGAACGAAAAGAAAAAGAGGAGGCATGAAAAAAGAAAAAGAAAAAGAAATAAATGACTTATATGACTTATATAAAAAAAACGTTTGTATGTGGAGAATTTATCTTATTTCTGCCTTCTTAAAATGTATGATTTCTAATAAAGAATTATCATGACCTTTTAGTATCTAATTAATTACTTGAATTTGCAATTAAACTAAATATTAAACCAAGTTAAACTTCATACATATAACTAGAATCAATCCAACACTGTCATATTTAACTTCTTTTAGATTTGCATAATTTTTTTTTTGTATAGGCTTTTTTTATTAGTTTCATACTGGAATTTTTTATTTTTTGGAGTTAATAAATCTAAATTTTTAAGTTATAAAAATTCTACTATATATCATAATATAAAATGATTTATTCTAAAAATTTAATTAATGCCAATGAGTTATAATTCAAATGACATAGTCTTTCTATACTTACTTAGAAATTGCGGGTTTAAATCTCTTTATCTTAGGTAAAAAAAAAATTGATAAATAAAAGAGACATAAATAATTATATCACTATCAAACTCAAGCAATAAATTTAGTTATTAATTTTGAGCGAGGATAAGTTATAAATTATGCTACAATGAACATACACCATTAATAGTTTTAATACTATTTTTAATTTCTTTAGGTTTATATTATATATGTTAATTAATAAACAAATAAAATTGTCTTCATCTTCTCCATCTTCTATTCTTCTCTCTATATATACTCACCCAACAAAATCTTGTTCCCCATCATCTTATATATATAAAAAGGAGGAAGTTTTTTTAAGCAAGTACTAGCTAGCCCTATATTCCTAATTTGGCCACACAACTATTATATATTCACATACATAAACAATTATTTCTTCTTTGGTTCTAAAATTTCTTCAACACC

>AhGA3ox5-p

TATGGCCCTATGATATGTTGTCTTATAGTGAATGAATGTGACCATAACCATTAACCGAGCTGGTGGAAGGGTTTGATTTTGATGTTATCCTCAGCTTCAAGACAAGACAATGGCACATCACATGTGTTAGTGTAGGCTTGTAGGGCTACTAAGGAAAAATTATTTTTTACTAGTTATTTATTTTGTTTTTAACGATAATAAAATAATCGCTAATATATTTATCGATAATTATATATTAATAATCTTATATTTTGTTATCAAAATTTTTTAGTAATAACTATATAATTATTAATAAATTTTTTTAATAATTACAAAAAATATAATTATTATTATAATATATAATAATAATAAATATATAATTATATATTGTAAAATATAAGTTAAATAAAACGTATAATAATTATTATTTAAATGCCACTAAAAGTAATTTTTATTGCAATAGAATTTATATTTTTTTCTTTTAAGTAACGTTTGTTTTGAGGTTCAGTATTATGTTTATTGATTTAGAGATTGATATTAAAATTTCAGTCTCTATTTCTAAAATTTCAGTATTTCAGTACTTTTAAAAAGTAGAGACAAAAGGAACTAAAATTTTTGAAGACGAAAACTGAAATTTTAATATTTTATAATTACTGTTATTACGTCATATATATTTCCTAGATTCCCTCTCAATTTTAATTTGTTATTCCAGTAATATATATATATATATATATATTATATATATAGTCGTTATAGTTTTTTTTCCCCTGAATATTTGGAGATCAGGAGGTATGAATAATTAGAGAGATTTCAATATATATATAATTATATAATTTGGATGTGATAAAATATTTTTAAAACTTGATTTACATGTACATATATAATTGAAATTCACAATTCTTTAATTTACACACTTTCCTAGCAAAATTCAACTCTTACATGATTGAAATGTCAAATTATTCATACATGATTCCACACACCTTTAAAAGGGTATCGCCATCAGTTTGTACCAATAAATAGTGAAGTTGGTTGTCCTTTCCTTTTTATATGTTTGTTCTTCCCTTTTTATAAAAATATTACGTGTATATTAAAAATTATTTATTAAATTATTTTATATATTTATTTATATATATACATATATTAATTCATACATTTTTAATATATGTATATTTATATAAGTGATTAATTTTTTTATGTACATATAACTAATTTTTGTATTTTTTTTTCAAAAACACTAATGTATCACAATATTTATTTGCACTCTTTATTTTTTAATAGAAGAAAATTATTTATTTTTTAAAAATTTAATGTAAAAGAAAAAAAATACCCCTACCTTAATTAAAAAGCACCGTAGCATATGGGGATCATTCATAGTGAAAGTTCAACAATAATAATTGACTATACTTTAAAGTAATAAGATTATTCTATTTTAAATATAACATGTGATATTTAAATATAGAAATCATAGAGTTTATTTCTGCCTTCTTAAATTGTATGATTTCTAATAAAGAATTACCATAACCTTTTAGTATCTAATTAATTACTTGAATTTGTAATCAACCTAAATATTAAACCAAGTTAAACTTCATACATATAACTAGAATCCAATCCAACATACACTCTCATATTTAAGCTTCTTTTAGATTTGCAATTTTTTTTTGTATAGGCTTTTTTTAGTTTCAGTGAAATTATTTTATTTTTTAGAGTTAATAAATCTAAATTTTTAAGTCATAAAAATTCTAATATCAAATTATAAAATAATTTATTCTAAAAATTTAAATTAATAAAAAAATACATAAATAATTATATATCACTATCAAACCCGAGCATAATAAATTTAGCTCTATTAATTTTGAGCCAGAATAAGTTATAAAATTATGCCACAATGAACCATTAATACTATTTTCTTTATGTTTATATTATATATGTTAATTAATAAACAAATAAAATTGTCTTCATCTTCTCCATCTTCTATTCTTCTCTTCTATATATACTCACCCAACAAAATCTTGTTCCCCATCTTATATATTTAAAAAGGAGTAAGTTTTTTCAAGCAAGTACTAGCTAGCCCTATATTCCTAATTTGGCCATACAACTATATATTATATATTCACATAAACACTTATTTCTTCTTTGGTTCTAAAATTTCTTCAACACC
