# Supplementary material for: Genome-wide identification and expression analysis of GA20ox and GA3ox genes during pod development in peanut
Source: PeerJ. 2023 Oct 26;11:e16279. doi: 10.7717/peerj.16279 (PMC10615029; doi:10.7717/peerj.16279)
Supplement: Table S4 [file peerj-11-16279-s004.docx]

**Table S4. key cis-acting elements of *AhGA20ox* and *AhGA3ox* genes**

| **Element name** | **Motif sequence** | **Function annotation** |
| --- | --- | --- |
| Box 4 | ATTAAT | part of a conserved DNA module involved in light responsiveness |
| ABRE | ACGTG/CACGTG/GCAACGTGTC/TACGTGTC/CGCACGTGTC | cis-acting element involved in the abscisic acid responsiveness |
| G-box | CACGTG/TACGTG/ACACGTGT/CACGTT/CACGTC/GCCACGTGGA/TAACACGTAG/CACGAC | cis-acting regulatory element involved in light responsiveness |
| TCT-motif | TCTTAC | part of a light responsive element |
| GA-motif | ATAGATAA | part of a light responsive element |
| GATA-motif | GATAGGA/AAGATAAGATT/AAGGATAAGG/GATAGGG | part of a light responsive element |
| AT1-motif | AATTATTTTTTATT | part of a light responsive module |
| TATC-box | TATCCCA | cis-acting element involved in gibberellin-responsiveness |
| MBS | CAACTG | MYB binding site involved in drought-inducibility |
| CAT-box | GCCACT | cis-acting regulatory element related to meristem expression |
| TGA-element | AACGAC | auxin-responsive element |
| LTR | CCGAAA | cis-acting element involved in low-temperature responsiveness |
| GT1-motif | GGTTAAT/GTGTGTGAA/GCGGTAATT/GGTTAA | light responsive element |
| TCA-element | CCATCTTTTT/TCAGAAGAGG | cis-acting element involved in salicylic acid responsiveness |
| GARE-motif | TCTGTTG | gibberellin-responsive element |
| P-box | CCTTTTG | gibberellin-responsive element |
| TC-rich repeats | GTTTTCTTAC/ATTCTCTAAC | cis-acting element involved in defense and stress responsiveness |
